# Evaluation of Transcatheter Pulmonary Valve Endocarditis by Dual-Energy Computed Tomography

**DOI:** 10.7759/cureus.8851

**Published:** 2020-06-26

**Authors:** Sarv Priya, Prashant Nagpal, Aditi Vidholia, Imroz Singh Sachdev, Ravi Ashwath

**Affiliations:** 1 Radiology, University of Iowa Hospitals & Clinics, Iowa City, USA; 2 Cardiothoracic Radiology, University of Iowa Hospitals & Clinics, Iowa City, USA; 3 Hematopathology, University of Iowa Hospitals & Clinics, Iowa City, USA; 4 Radiology, University Hospital of North Durham, Durham, GBR; 5 Pediatric Cardiology, University of Iowa Stead Family Children's Hospital, Iowa City, USA

**Keywords:** transcatheter pulmonary valve implantation, transcatheter pulmonary valve endocarditis, cta, dual-energy cta, virtual monoenergetic images, iodine perfusion map, rvot, tpvi, melody valve

## Abstract

Transcatheter pulmonary valve implantation (TPVI) is now an established alternative to surgery in patients with congenital heart disease and dysfunctional right ventricular outflow tract (RVOT) conduit. However, there is recognition of a higher incidence of infective endocarditis in the patients after TPVI. Transthoracic and transesophageal echocardiography is limited in the evaluation of prosthetic pulmonary valve endocarditis secondary to a metallic artifact and degenerative calcified conduit. Additionally, the anterior position of RVOT also limits evaluation by echocardiography. Conventional single-energy CTA can also be sub-optimal in evaluating prosthetic pulmonary valve stent frame due to streak artifacts from the metallic cage and poor contrast to noise ratio if higher kV is used for single-energy CTA to avoid metallic artifacts. Dual-energy CTA can overcome these limitations using reconstructed virtual monoenergetic and iodine-only images for metal artifact reduction and improve intra-stent luminal visualization. Reconstructed iodine perfusion maps may also help differentiate vegetation from a thrombus. In this case report, we discuss the diagnostic utility of dual-energy cardiac CT in the evaluation of endocarditis after TPVI and discuss the imaging protocol.

## Introduction

Transcatheter pulmonary valve implantation (TPVI) is an established alternative to surgery for patients with dysfunctional (stenotic/regurgitant) right ventricular outflow tract (RVOT) conduit [1]. However, patients who receive TPVI have a higher incidence of infective endocarditis, which needs early detection and management to prevent complications. Both transthoracic echocardiography (TTE) and transesophageal echocardiography (TEE) are challenging in these cases due to incomplete assessment of the RVOT conduit secondary to the anterior position of RVOT, a metallic artifact from the stent frame, and calcific degenerative changes in the conduit [2]. Conventional single-energy CTA is also limited in the evaluation of endocarditis after TPVI secondary to beam hardening and dense streak artifacts. Dual-energy CTA (DECT) can overcome these limitations using reconstructed virtual monoenergetic and iodine-only images to improve visualization of the prosthetic pulmonary valve. CT may improve diagnostic evaluation in patients with prosthetic valves as outlined in the European Society of Cardiology (ESC) 2015 guidelines [3].

The use of dual-energy CTA is steadily increasing in the field of cardiovascular applications like coronary plaque evaluation and myocardial perfusion. These have been made possible due to significant improvement in temporal resolution and superior spectral separation using tin filter at high kVp image [4-5].

Despite these advantages, the role of DECT in the evaluation of endocarditis after TPVI has not been studied earlier. Using case examples, we discuss our initial experience in the challenging clinical situation of suspected endocarditis after TPVI.

## Case presentation

Case 1

**Figure 1:**
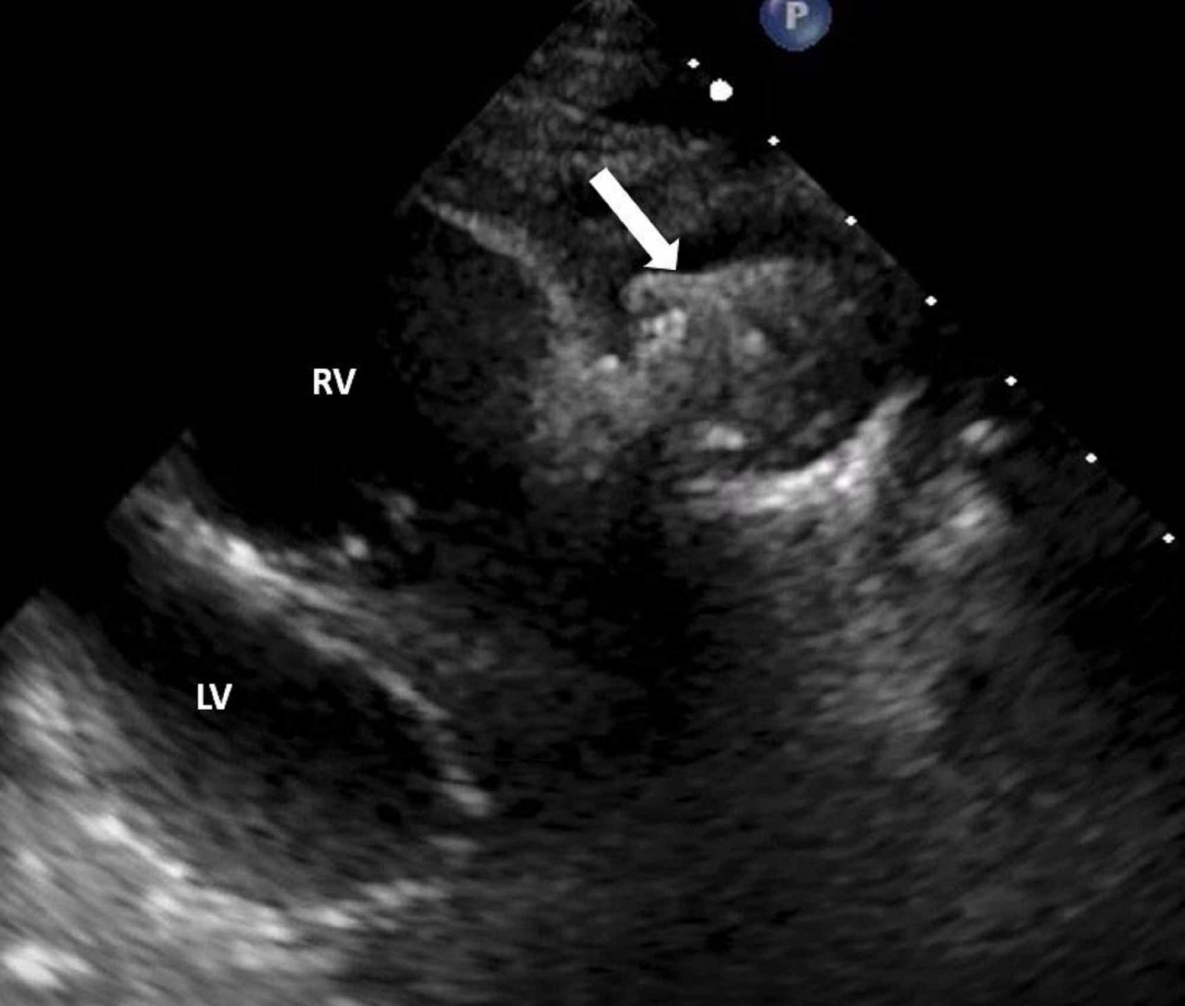
TTE in a 17-year-old patient with transcatheter pulmonary valve and features suspicious of infective endocarditis A still image from TTE shows suboptimal evaluation of the right ventricular outflow tract due to artifact from the transcatheter pulmonary valve (Melody valve) stent apparatus (arrow). RV, Right ventricle; LV, Left ventricle; TTE, Transthoracic echocardiogram

A 16-year-old female with a history of Tetralogy of Fallot and TPVI (Melody, Medtronic Inc, Minneapolis, MN) presented with fever three years later. Blood culture was positive for methicillin-sensitive *Staphylococcus aureus* (MSSA). TTE was suboptimal (Figure 1) due to metallic artifact but showed an increased peak (51 mm Hg) Doppler gradient across the stent without an apparent cause.

**Figure 2:**
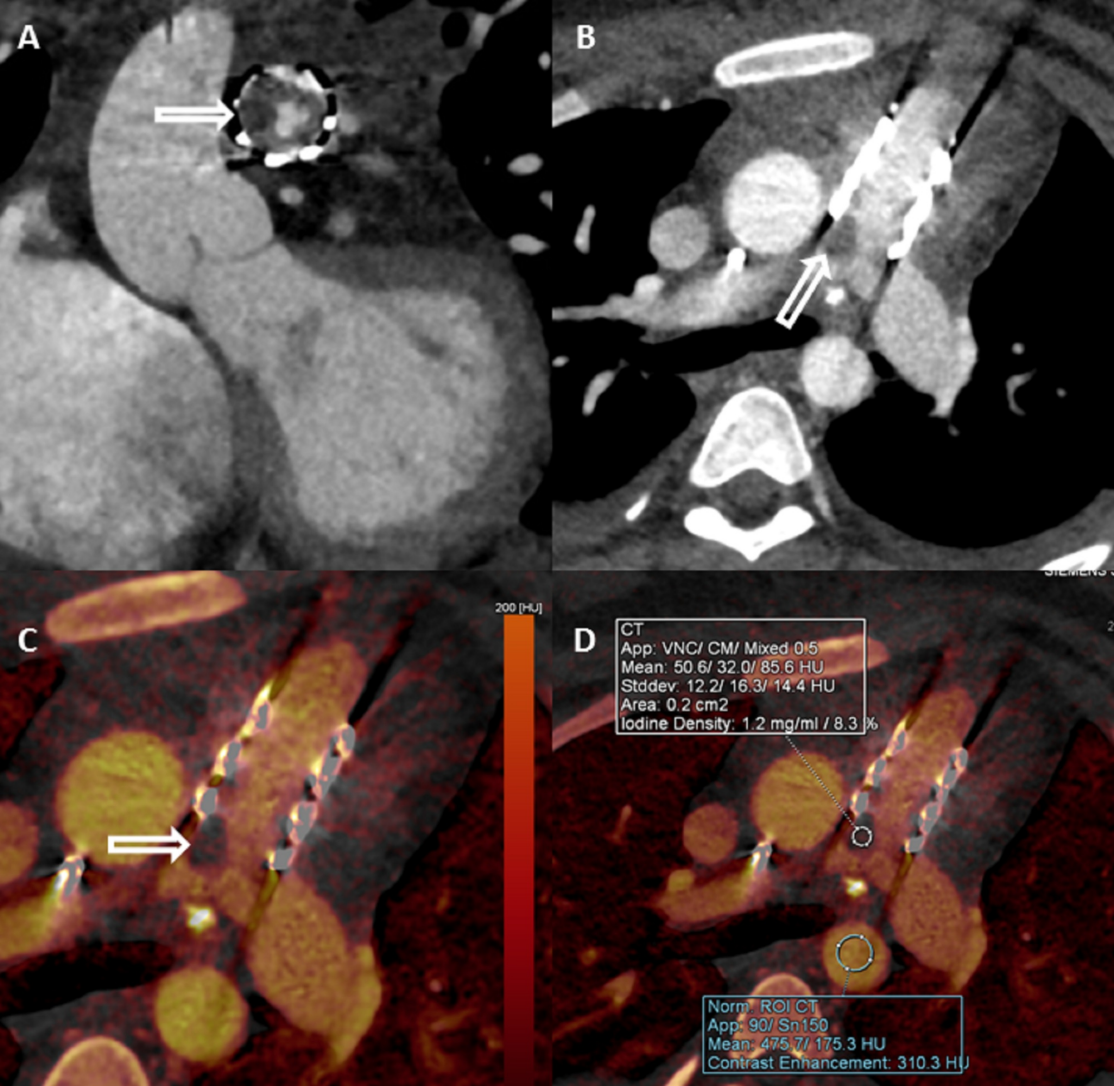
ECG-triggered multiplanar reformatted dual-energy CTA using dual-source CT at 90 kVp/150Sn kVp in a 17-year-old female with infective endocarditis (Case 1) Reformatted coronal (A) and axial (B) linear blended (50 % of each single energy) CTA images showing a circumferential filling defect in transcatheter pulmonary valve stent frame (arrow). Iodine perfusion map images show the presence of iodine uptake (arrow in C) within the filling defect, quantified as (1.2 mgI/ml) (D) suggesting the presence of infection. ECG, Electrocardiogram; CTA, Computed tomography angiography

DECT (DLP 201.6 mGycm) showed circumferential hypodensity in the stent frame (Figure 2A, 2B). Quantitative iodine uptake (1.2 mg/mL) was seen within this hypodensity, confirming infective vegetation over bland thrombus (Figure 2C, 2D).

**Figure 3:**
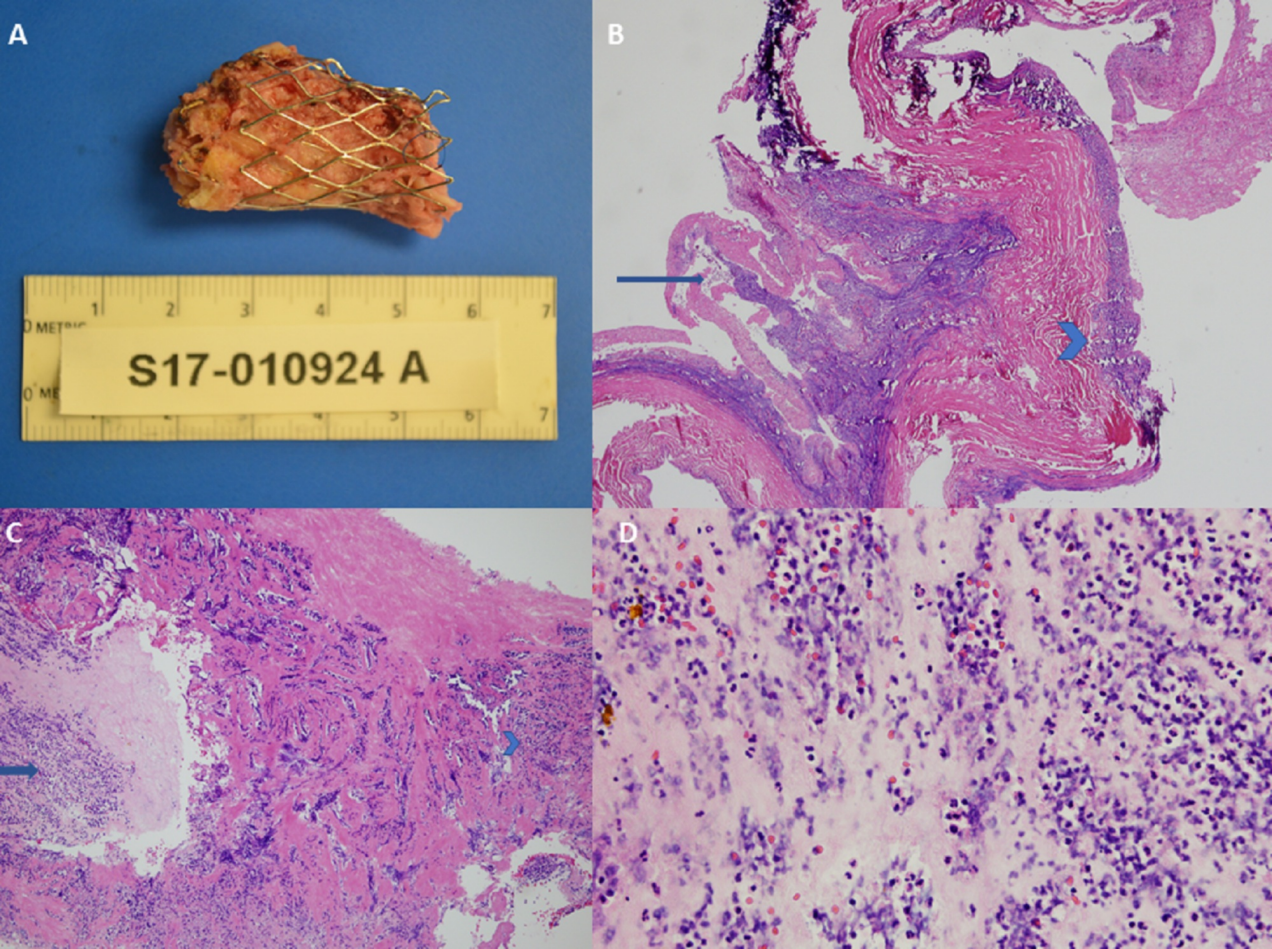
Gross and microscopic histopathologic examination of explanted transcatheter pulmonary valve (Case 1) Gross explanted specimen (A) of transcatheter pulmonary stent frame is seen. Microscopic examination (B-D) shows the presence of fibrinopurulent exudate (blue arrow). Arrowhead (B, C) shows valve tissue with acute inflammation.

The patient underwent surgical replacement with a homograft. Histopathology of explanted conduit (Figure 3A) confirmed acute inflammation with fibrinous exudate forming vegetation (Figure 3B, 3C, 3D).

Case 2

**Figure 4:**
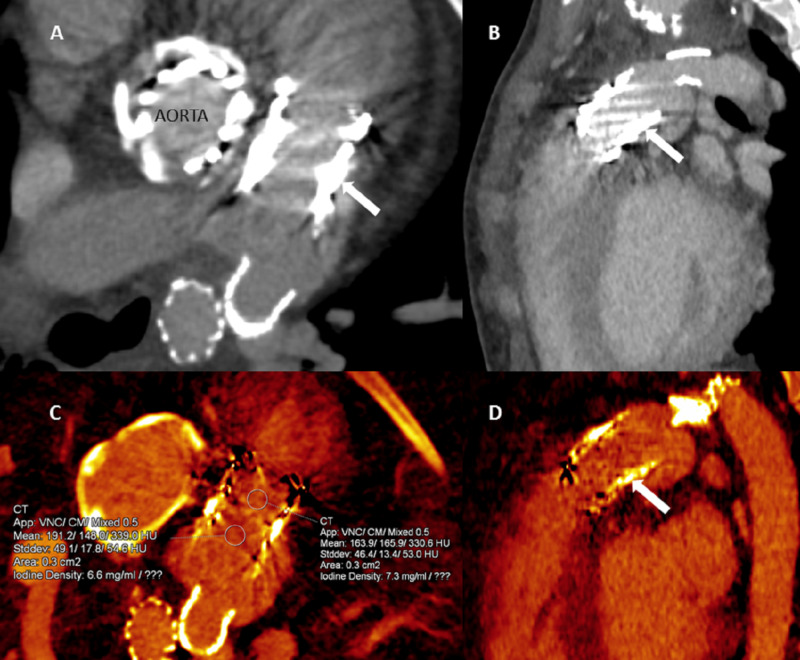
ECG-triggered dual-energy CTA images in a 16-year old female with infective endocarditis using dual-source CT at 90 kVp and 150 kVp (Case 2) Linear blended (50 % of each single-energy) CTA axial (A) and oblique sagittal (B) images show severe streak artifact from pulmonary valve stent frame with obscuration of the lumen (arrow). Prosthetic aortic valve (A) is also seen. Reconstructed Iodine quantitative (C) and qualitative (D) perfusion maps show a homogeneous distribution of iodine within the stent lumen (arrow) and uptake greater than 0.5 mgI/ml; suggesting true enhancement with complete contrast opacification within the stent frame. CTA, Computed tomography angiography; ECG, Electrocardiogram

A 16-year-old female with TPVI (Melody, Medtronic Inc, Minneapolis, MN) for congenital stenosis presented with a fever a month later and positive blood culture for gram-positive cocci. Transthoracic echocardiogram was non-diagnostic. DECT was ordered to evaluate the stent frame integrity and to rule out any pseudoaneurysm. DECT (DLP 156.2 mGycm) with blended single-energy images showed metallic artifacts obscuring the lumen (Figure 4 A, B). Reconstructed iodine maps showed the diffuse distribution of iodine within the stent lumen, suggesting uniform opacification of the stent lumen and ruled out evidence of significant thrombus/vegetation (Figure 4 C, D).

No other complications like infective pseudoaneurysm or dehiscence were seen. She was managed conservatively using IV antibiotics and was discharged in a stable condition after two weeks. The patient was clinically afebrile and asymptomatic at a three-month follow-up evaluation.

Cardiac DECT Technique

The cases were performed on a dual-source CT scanner (Siemens Drive, Erlangen, Germany) with a tube voltage of 90 and 150 kVp and automated tube current modulation (Caredose 4D). ECG-gating with dose modulation was performed. Scan range extended from the level of carina to the diaphragm.

Injection Technique

A biventricular or triphasic injection protocol was employed for the simultaneous evaluation of aorta and pulmonary artery. In this technique, the half dose of total contrast volume (Iohexol 350 mgI/ml) was followed by the remaining contrast volume mixed with saline (50:50) and saline chase flush. This method is commonly used in patients where the evaluation of both right- and left-sided cardiac chambers is required. The scan was triggered using bolus tracking (descending aorta at 100 HU), providing adequate opacification of cardiac chambers and RVOT conduit.

Image Reconstruction

**Figure 5:**
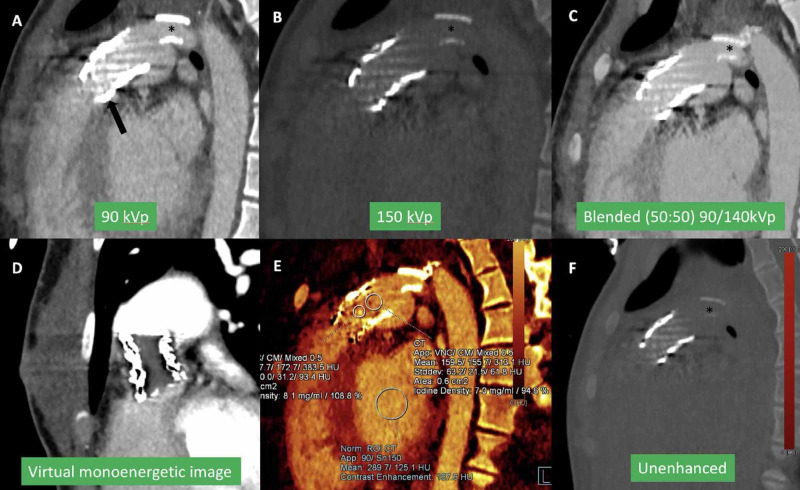
Type of CT data sets reconstructed from dual-energy CTA Single energy 90 kVp (A) and 150 kVp (B) images with mixed weighted images (50% weighting of each single energy), also known as linear blended images that simulate single energy 120 kVp images (C).  Iodine perfusion maps (D, E) with quantitative iodine uptake (E). Virtual unenhanced CT image after subtracting iodine (F). CTA, Computed tomography angiography

Post-processing was performed on Siemens Syngovia software. The CT images were reconstructed over 10 phases per cardiac cycle for dynamic valve and functional analysis. Six data sets were reconstructed: single-energy data set at 90 kVp (Figure 5A) and 150 kVp (Figure 5B), mixed weighted average image (blended) simulating 120 kVp single-energy CTA (Figure 5C), virtual monoenergetic image (Figure 5D), iodine map (Figure 5E), and virtual unenhanced CT (Figure 5F). These data set reconstructions can be performed from acquired raw data and do not result in added radiation exposure.

Virtual Monoenergetic Images

**Figure 6:**
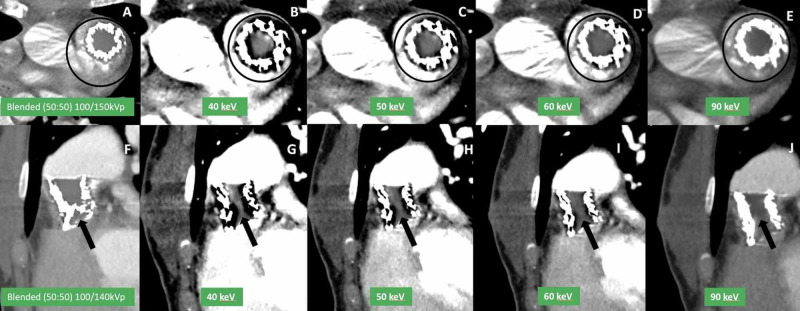
ECG-triggered dual-energy multiplanar coronal CTA using dual-source CT at 90 kVp/150 Sn kVp in a patient with endocarditis with virtual monoenergetic image reconstruction Linear blended (50% of each single energy) CTA axial (A) and oblique sagittal (F) images show suboptimal evaluation of pulmonary artery lumen due to poor contrast opacification within RVOT stent lumen (circle in A, arrow in F). Virtual monoenergetic images reconstructed from dual-energy data show significant improvement in visualization of intra-stent lumen (circle B-E; and arrow G-J) with best opacification at low keV (40/50 keV) images.

Virtual monoenergetic (VM) images were reconstructed, ranging from 40 to 190 keV. High keV data were useful for evaluating the structural integrity of the stent frame, while low keV (40-70) images improved RVOT contrast enhancement (Figure 6) [6]. Virtual monoenergetic images simulate as if the image was acquired by a single X-ray photon energy in contrast to the polychromatic X-ray photon energy spectrum of conventional CT, thereby resulting in maximum photon attenuation and contrast specific to the energy chosen. VM images provide an optimum balance of increased contrast attenuation at low keV and decreased image noise at high keV with increased conspicuity in detecting prosthetic vegetations or thrombus. VM images also help in salvaging studies with poor contrast opacification due to suboptimal contrast bolus and timing.

Virtual Non-Contrast Images

Virtual non-contrast images are reconstructed by subtraction of the iodine from contrast-enhanced images; hence providing an artificially created non-contrast images. These images are useful for examining prosthetic material and post-surgical changes. This prevented performing an unenhanced CT, thus reducing radiation exposure [7].

Iodine Images

**Figure 7:**
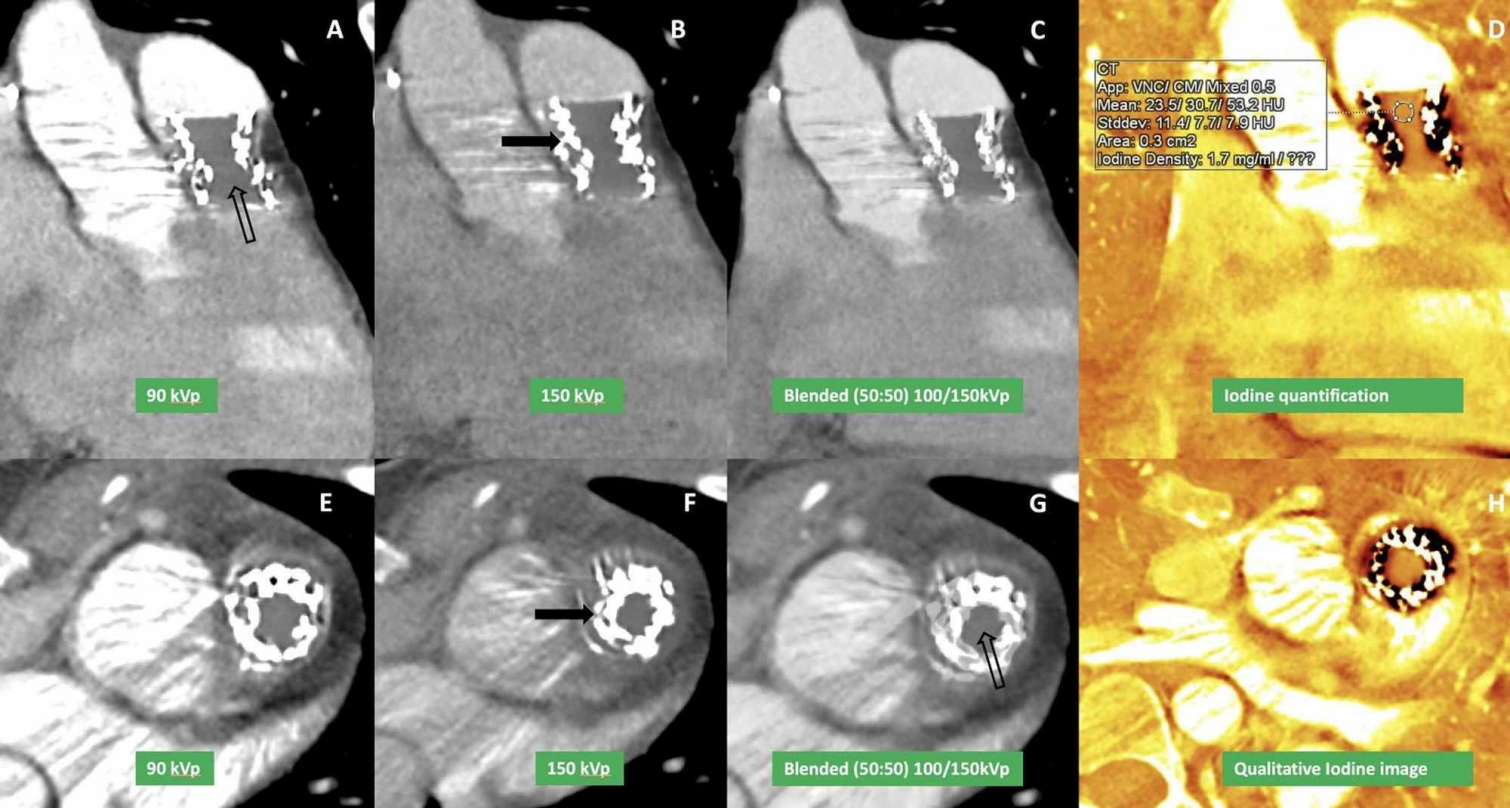
ECG-triggered dual-energy multiplanar coronal CTA using dual-source CT at 90 kVp/150 Sn kVp in a patient with endocarditis with iodine perfusion image reconstruction Single-energy coronal and axial images at 90 kVp (A, E) and 150 kVp (B, F) with linear blended (50% of each single-energy) CTA images (C) show poor opacification of the stent lumen (arrow). Reconstructed iodine perfusion images show significant improvement in the assessment of stent lumen with uniform iodine presence (D, H) suggesting diffuse contrast opacification of the lumen. ECG, Electrocardiogram; CTA, Computed tomography angiography

Iodine-only images involve accentuation of the iodine attenuation by post-processing. Iodine-only images improved visualization of the intrastent lumen by avoiding metallic artifacts (Figure 7) and enabled quantification of the iodine uptake within the abnormality seen in the valve.

**Figure 8:**
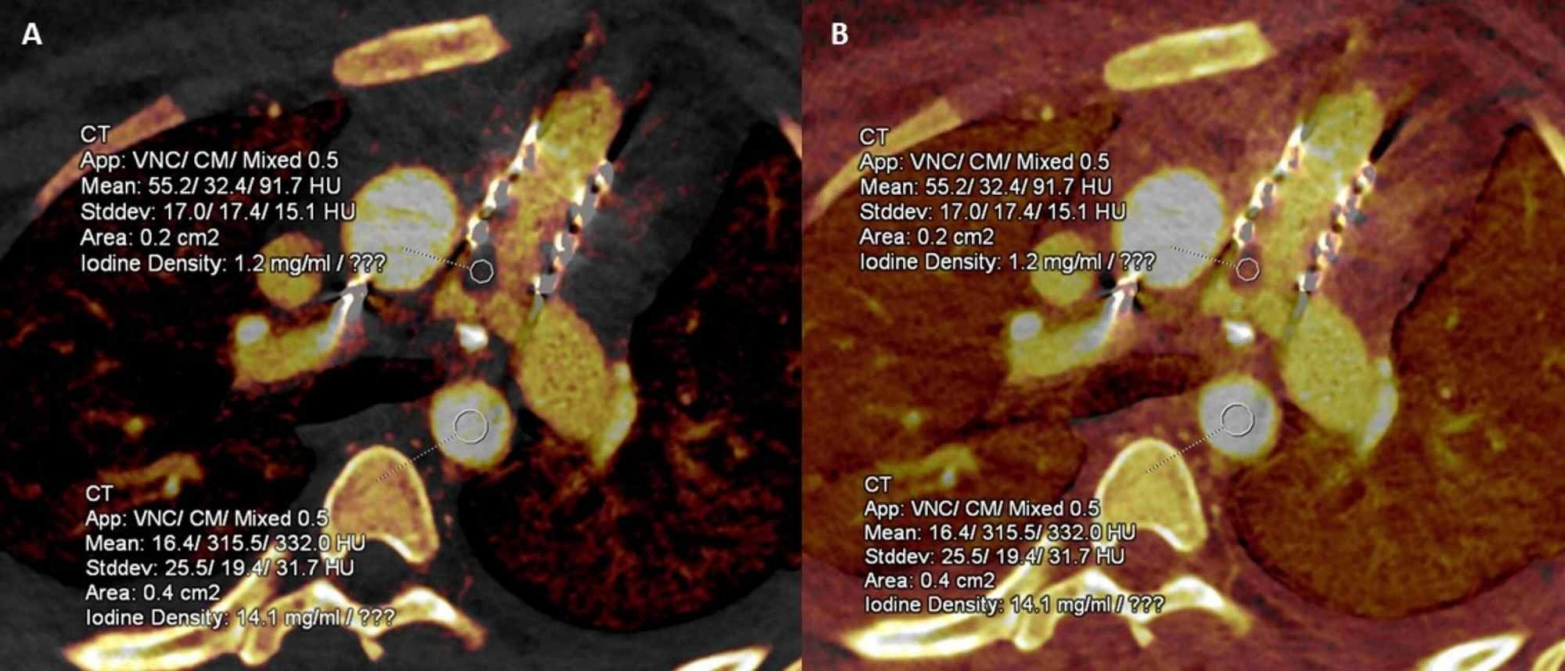
Iodine quantitative perfusion map images in a patient with infective endocarditis Iodine uptake perfusion map images show iodine quantification (A) within filling defect in the pulmonary artery prosthetic stent frame (1.2 mgI/ml). After changing the contrast window, no change in iodine uptake is seen (B) suggesting that iodine uptake is not affected by window level. Also shown is uniform iodine uptake in descending aorta (larger circle).

Iodine uptake is useful to differentiate vegetation from a thrombus (Case 1) and is not affected by window-level (Figure 8).

## Discussion

TPVI is FDA approved (since 2010) for patients with dysfunctional RVOT conduit [8]. The annualized rates of endocarditis after TPVI range between 3% and 4.2% [9, 10]. Early diagnosis is critical for timely management. Echocardiogram, including intracardiac echocardiography, may be insufficient for evaluation due to metal artifact obscuring valve leaflets [11]. MRI is also limited due to susceptibility artifact from the stent frame [12]. The recent European Society of Cardiology (ESC) 2015 guidelines have advocated additional use of cardiac CT, positron emission computed tomography (PET-CT), and single-photon energy computed tomography (SPECT-CT) in patients with prosthetic valve endocarditis [3]. However, concerns about false-positive and false-negative examinations limit the use of PET-CT [13]. Besides, SPECT-CT is time-consuming and has low spatial resolution with low count detection relative to PET-CT, restricting its widespread use.

The choice of test depends upon the goal of the study and the clinical status of the patient. A transthoracic echocardiogram is the initial test of choice for the management and monitoring of these patients. It provides a quick, bed-side assessment of cardiac function in unstable patients. However, both transthoracic and transesophageal echocardiograms are frequently limited in the evaluation of endocarditis affecting intracardiac devices or prosthetic valves [3]. Cardiac MRI is an excellent non-invasive modality for evaluating the ventricle function. However, direct visualization of the thrombus/vegetation and assessment of the degree of pulmonary insufficiency is limited due to artifacts from the stent frame in these patients. ^F-18 ^FDG PET-CT or white cell-labeled SPECT-CT are commonly used to evaluate for the source of infection when other modalities, including echocardiogram/computed tomography, fail to localize the site of endocarditis. PET-CT is more sensitive but has low specificity, whereas SPECT-CT has high specificity in endocarditis evaluation [14]. CTA should be performed in patients with suspected endocarditis after TPVI, where echocardiogram is limited in information regarding the cause of rising Doppler gradient across the pulmonary valve. Additionally, CTA provides a complete assessment of paravalvular complications such as pseudoaneurysm, abscess, fistula, vessel rupture, and pulmonary septic emboli and is invaluable in pre-operative surgical planning including coronary artery evaluation [3].

Conventional single energy CT in patients with metallic prosthetic valve evaluation is usually limited due to streak artifacts. Dual-energy CT angiography (DE-CTA) can overcome these limitations and has distinct, complementary advantages like virtual monoenergetic images, metal artifact reduction, and iodine quantification, which allows for better visualization and appropriate disease characterization in such patients. CT is fast, non-invasive, and is also excellent in detecting paravalvular complications of prosthetic valve endocarditis. The radiation dose with single-energy and dual-energy cardiac CT is comparable [15, 16]. The use of virtual non-contrast also obviates the need for a non-contrast run, as with single-energy scans.

Unlike single-energy CTA, DECT may help differentiate thrombus from infective vegetation based on the presence or absence of iodine uptake. We found iodine uptake in the filling defect in Case 1 (1.2 mgI/ml), and it was confirmed as infected vegetation on histopathology. Prior studies have proposed that iodine uptake at a concentration above 0.5 mgI/ml suggests true enhancement [17]. Currently, there is no available literature regarding the use of this technique in this clinically challenging situation where other imaging techniques are non-diagnostic or non-specific. Cardiac DECT technique is promising to visualize the integrity of the prosthetic valve as well as surrounding cardiac structures and identify evidence of infection. This needs to be validated in a larger sample size and future multicenter studies.

## Conclusions

Endocarditis after TPVI is a high-risk situation requiring early diagnosis and treatment. Echocardiography is frequently suboptimal in these patients due to the metallic valve frame. Conventional single-energy CT in these patients is also poor due to streak artifacts from the metallic valve cage. Cardiac DECT enables reconstructions like virtual monoenergetic and iodine-only images that overcome the limitations of single-energy CTA. Quantitative iodine uptake is also possible with DECT, which allows differentiation of a bland thrombus versus infective vegetation improving the specificity of the diagnosis. The use of DECT for assessment of endocarditis after TPVI is a new and novel use of CT and should be validated in future studies.
